# Cardiovascular disease lifestyle risk factors in people with psychosis: a cross-sectional study

**DOI:** 10.1186/s12889-018-5649-5

**Published:** 2018-06-15

**Authors:** Doreen Mucheru, Mary-Claire Hanlon, Linda E. Campbell, Mark McEvoy, Lesley MacDonald-Wicks

**Affiliations:** 1 Faculty Health and Medicine, The University of Newcastle, Callaghan, 2308 Australia; 2 Brain and Mental Health Program, Hunter Medical Research Institute, New Lambton, 2305 Australia; 3 Priority Research Centre for Brain and Mental Health, The University of Newcastle, Callaghan, 2308 Australia; 4 School of Medicine and Public Health, The University of Newcastle, Callaghan, 2308 Australia; 5 Faculty of Science, The University of Newcastle, Callaghan, 2308 Australia; 6 Priority Research Centre GrowUpWell, The University of Newcastle, Callaghan, 2308 Australia; 7 Centre for Clinical Epidemiology & Biostatistics, Hunter Medical Research Institute, New Lambton, 2305 Australia; 8 Priority Research Centre for Physical Activity and Nutrition, The University of Newcastle, Callaghan, 2308 Australia

**Keywords:** Psychosis, Nutrition, Physical activity, Smoking, Lifestyle risk factors, Cardiovascular disease, Lifestyle interventions

## Abstract

**Background:**

People with psychosis die on average 25 years earlier than those in the general population, with cardiovascular disease (CVD) contributing to much of the excess mortality. This cross-sectional study aimed to identify the relationship between lifestyle risk factors for CVD – poor nutrition, smoking and low physical activity levels – and dyslipidaemia, hypertension and hyperglycaemia while controlling for potential confounders in 1825 people from the Survey of High Impact Psychosis (SHIP) in Australia. We also aimed to identify clustering patterns of lifestyle risk factors and associated demographic variables.

**Methods:**

Three logistic regressions were used to predict the effect of nutrition, smoking and physical activity on dyslipidaemia, hypertension and hyperglycaemia while controlling for clozapine use, sex and age. Clustering patterns of nutrition, smoking and physical activity were examined using the two-step cluster method which is based on hierarchical cluster analysis. Demographic variables associated with different clusters were identified using measures of association.

**Results:**

Smoking status had a positive association with dyslipidaemia (adjusted odds ratio = 0.50; 95% confidence interval = 0.32–0.78; *p* = 0.002). Other cardiovascular disease lifestyle risk factors did not have a significant relationship with dyslipidaemia, hypertension and hyperglycaemia. Clustering patterns of lifestyle risk factors showed that younger men, with low education levels, and relying on a government pension, were most likely to display the poorest lifestyle risk behaviours. The largest cluster (42%) of participants was characterised by a mixed demographic profile and were most likely to display poor nutrition and low physical activity levels but less likely to smoke.

**Conclusions:**

Only smoking status had a significant positive association with dyslipidaemia which could indicate that there are additional factors affecting the relationship between other cardiovascular lifestyle risk factors and dyslipidaemia, hypertension and hyperglycaemia in people with psychosis. Unknown confounders and traditional lifestyle risk factors may explain the high rates of CVD in this group. Clustering of lifestyle risk factors and their demographic profiles could help the design of intervention programs in people with psychosis.

## Background

Cardiovascular disease (CVD) is the collective term for all conditions which affect heart and blood vessels [1]. In 2016, CVD was the leading cause of death in 190 countries around the world and on average claims about 17.3 million lives per year with this number expected to rise to more than 23.6 million by 2030 [2]. CVD is a prominent comorbidity among people with psychosis, contributing to much of the excess mortality in this group; worldwide estimates of mortality show people with psychosis die, on average, 25 years earlier than those in the general population [3, 4].

CVD is exceedingly prevalent in people with psychosis because of the high prevalence of risk factors [5]. Non-modifiable CVD risk factors are inborn and include having a family history of the condition, increasing age, ethnicity and male gender [6]. Modifiable risk factors for CVD are subject to change and constitute dyslipidaemia, hypertension, hyperglycaemia, inadequate physical activity, poor nutrition, smoking, central obesity, overweight (body mass index [BMI] of ≥25) and obesity (BMI ≥ 30) [7, 8]. Dyslipidaemia constitutes abnormally raised total or low-density lipoprotein (LDL) cholesterol or low high-density lipoprotein (HDL) cholesterol [9]. Hypertension is elevated blood pressure that is above established cut-off values while hyperglycaemia is raised blood glucose that is also above reference ranges [10, 11]. The National Health and Medical Research Council (NHMRC) considers nutrition poor if food intake is inconsistent with dietary guidelines and also defines physical activity as inadequate when one displays insufficient activity levels based on recommendations of minimum activity [12, 13]. A waist circumference of > 80 cm in women and > 94 cm in men is associated with increased risk of chronic disease and is known as central adiposity [8]. Atypical antipsychotic medications, the cornerstone of treatment in psychosis, also increase CVD risk by exacerbating weight gain, hyperglycaemia and dyslipidaemia [14].

The Survey of High Impact Psychosis (SHIP), Australia’s second national psychosis survey, reported a high prevalence of modifiable CVD risk factors; more than half of the SHIP cohort (54%) presented with metabolic syndrome – a combination of hypertension, dyslipidaemia, hyperglycaemia and central obesity [15, 16]. Dyslipidaemia, hypertension and hyperglycaemia are a product of poor nutrition, smoking and inadequate physical activity, collectively known as lifestyle risk factors [17]. Pharmacological management of dyslipidaemia, hypertension and hyperglycaemia only partially decrease the risk of CVD in the presence of lifestyle risk factors [17]. The Diabetes Prevention Program research group found that metformin decreased the risk of diabetes almost twice as much in the group also receiving a lifestyle intervention [18]. In a prospective cohort study of 42,847 otherwise-healthy men taking medications for hypertension or hypercholesterolemia, 62% of deaths from coronary heart disease could have been prevented by adhering to dietary guidelines, not smoking and engaging in at least 30 min of physical activity per day [19]. Targeting lifestyle risk factors should therefore be central in CVD prevention [17].

Recently, questions have arisen as to what degree CVD lifestyle risk factors impact dyslipidaemia, hypertension and hyperglycaemia among people with psychosis [5, 20–22]. This is because people with psychosis have a 78% higher risk of developing CVD than the general population and develop the condition two decades earlier [23]. Having a psychotic disorder, in addition to medical management of psychosis, may confound the relationship between lifestyle risk factors and dyslipidaemia, hypertension and hyperglycaemia, which may increase the risk of CVD [21, 22, 24, 25]. Researchers in this field found that age, obesity, sedentary behaviour, family history of diabetes, treated hypertension and treated hypercholesterolemia show significant associations with current type 2 diabetes status among people with psychosis, however, authors acknowledge that additional unknown factors may further explain this relationship [26, 27]. Morgan, McGrath [24] and colleagues examined modifiable lifestyle risk factors with respect to metabolic syndrome and found that sedentary behaviour, overweight and obesity and smoking status all contribute to the metabolic syndrome among people with psychosis. Our research will add to this body of work by assessing the interplay of lifestyle risk factors with dyslipidaemia, hypertension and hyperglycaemia, whilst controlling for confounders [17].

In the general population, lifestyle risk factors for CVD tend to present in clusters [28]. Some suggest that a cluster of lifestyle risk factors for CVD should be treated as a single entity in intervention programs among groups likely to exhibit a cluster of all three risk factors [28]. This is because these interventions tend to be more effective and efficient at achieving intervention goals and minimize economic impact of lifestyle diseases [29–31]. People with psychosis are an ideal group to assess clustering of CVD lifestyle risk factors due to the high prevalence of the individual risk factors in this population [5, 15, 32, 33]. Correlates of physical activity and low fruit and vegetable intake – a measure of nutrition quality – have been identified in people with psychosis [32, 33]. The current paper will further this knowledge by identifying co-occurring patterns of all three lifestyle risk factors, and describe the demographic profiles of those presenting with different risk factor clusters which will be useful for the design, delivery and evaluation of lifestyle intervention programs in people with psychosis [5, 34, 35].

This study aims to describe the relationship between lifestyle risk factors for CVD – poor nutrition, smoking and low physical activity levels – and dyslipidaemia, hypertension and hyperglycaemia in a large cohort of men and women participating in SHIP, while controlling for potential confounding factors. Further, we identify clustering patterns of lifestyle risk factors in study participants, and describe the demographic characteristics associated with different clusters of lifestyle risk factors.

## Methods

This is a cross-sectional analysis of data from Australian adults living with psychosis who participated in SHIP.

### The survey of high impact psychosis (SHIP)

SHIP was conducted in seven catchment sites across Australia, which covered about 62,000km^2^ with a total resident population of about 1.5 million of those aged 18 to 64 years. This is equivalent to almost 10% of the Australian population within the same age bracket.

The comprehensive SHIP methodology has been provided elsewhere [15, 24]. In summary, participants were considered for study inclusion if they resided in one of the seven study catchment sites and were also in contact with public specialised mental health services (MHSs) or community-managed organisations (CMOs; formerly non-government organisations) funded to support people with mental illnesses. A two-phase sampling technique was utilised to identify participants for study inclusion. Phase 1 involved a census of those in contact with MHSs and CMOs supporting people with mental illness in March 2010 as well those in contact with public MHSs 11 months prior to March 2010. A psychosis screener was used to identify psychosis-positive individuals who were then randomly selected to take part in Phase 2 of the study. In Phase 2, participants were stratified by age group and underwent a diagnostic interview, fasting blood tests, physical checks and cognitive functioning tests. Stratification by age group was to ensure adequate coverage of younger as well as older participants. Participants were excluded from the study if they resided in a nursing home or prison, or were incapable of communicating due to a language barrier or intellectual incapacity. A total of 1825 participants were included in Phase 2 and were thus part of the final sample included in SHIP.

### Measures

#### Demographics

Demographic variables included: sex (male/female), age in years, Aboriginal or Torres Strait Islander descent (yes/no), current marital status (single or never married/married or de facto/separated or divorced/widowed), government pension as the main source of income (yes/no), highest qualification obtained (left school with no qualifications/secondary school qualification or leaving certificate/tertiary certificate/bachelor’s degree/postgraduate qualifications/other), income per fortnight (AU$300 or less/AU$300-AU$499/AU$500-AU$799/AU$800-AU$1000/AU$1000 or more) and engagement in paid employment in the last 12 months (yes/no).

#### Diagnosis

The Diagnostic Module of the Diagnostic Interview for Psychosis (DIP-DM) was used to diagnostically describe the sample [36]. DIP-DM responses were scored and entered into a computer algorithm that employed the Operational Criteria for Psychosis (OPCRIT) to produce an International Statistical Classification of Diseases and Related Health Problems 10th Revision (ICD-10) diagnosis thus reducing the likelihood of subjective bias [36, 37]. The DIP-DM has good inter-rater reliability (kappa value of ≥ 0.6) and excellent diagnostic validity (90% consistency with the Schedules for Clinical Assessment in Neuropsychiatry) [36].

#### Nutrition

Retrospective responses for fruit and vegetable intake were used as measures of diet quality via items from Short Diet Questions used in the 1995 National Nutrition Survey (NNS) [38]. Fruit and vegetable intakes were measured separately for the four-week period preceding SHIP, using self-report questions [39]. Validity of 1995 NNS questions measuring fruit and vegetable intake when compared against 24-h recall data, were fair [39]. Validity assessments showed that those who reported consuming a greater number of serves of fruit and vegetables also had higher intake as per 24-h recall data; this information did not however correlate with standard serve estimates, which are the recommended portions, as serve sizes tend to be overestimated by those using the tool [39].

The Australian Dietary Guidelines (ADGs) developed by NHMRC were used as the benchmark for either meeting or not meeting fruit and vegetable recommendations [13]. The ADGs recommend consuming five serves of vegetables and two serves of fruit a day [13]. For the purposes of our analyses, fruit intake and vegetable intake scales were converted into dichotomous variables comprising ‘met guidelines’ and ‘did not meet guidelines’.

#### Smoking dependence

Smoking dependence was measured using the Fagerstrom Test for Nicotine Dependence (FTND); a six-item tool used to measure addiction to smoking [40]. The FTND has acceptable test–retest reliability (*r* = 0.65) in the measurement of smoking behaviour in people with psychosis [40]. The FTND scores were categorized into outcome categories ranging from non-smoking to high dependence [41].

#### Physical activity

Physical activity was measured using the International Physical Activity Questionnaire (IPAQ), which gives a retrospective self-reported measure of time in minutes spent performing moderate or vigorous activity in the 7 days prior to interview. This tool is validated in people with schizophrenia with a criterion validity of 0.37 which is similar to that of the general population [42].

#### Metabolic health and blood pressure

Fasting blood samples were obtained from consenting participants and analysed by an experienced hospital scientist to evaluate levels for total cholesterol, HDL-C, LDL-C and fasting blood glucose. Physical measurements inclusive of resting blood pressure were also obtained from participants. Metabolic health measures were evaluated against National Heart Foundation (NHF) targets [9]. For the purpose of this study, dyslipidaemia was indicated if total cholesterol was ≥4 mmol/L or HDL-C was ≤1 mmol/L or LDL-C was ≥2.5 mmol/L [9]. Hyperglycaemia was determined if fasting blood glucose was ≥5.6 mmol/L [16]. Finally, hypertension was indicated if systolic blood pressure was ≥140 mmHg or diastolic blood pressure was ≥90 mmHg [11].

### Measurement of potential confounding variables

Based on previous literature, the following variables were tested for confounding: age, sex, psychotropic medications, diagnosis, education, income and employment [24–26]. Additional potential confounders were considered variables which, according to logistic regression analysis, were associated with the dependent variable at p level of < 0.2. Variables meeting this criterion were: clozapine use based on self-reported medications used for a period of ≥ 4 weeks in the 4 weeks preceding interview, sex and age.

### Statistical analysis

Statistical tests were conducted using the Statistical Package for the Social Sciences version 23 (SPSS). Descriptive statistics were performed for all variables.

Three logistic regressions were used to predict the effect of nutrition, smoking and physical activity on i) dyslipidaemia ii) hypertension and iii) hyperglycaemia. The models were controlled for the confounding variables, clozapine use, sex and age.

Clustering of nutrition, smoking and physical activity behaviour were examined using the two-step cluster method - a mode of hierarchical cluster analysis - to assess whether lifestyle risk factor patterns were evident [43, 44]. The two-step cluster method was used for the analysis because a large number of cases can effectively be processed (*n* > 1000 is considered large for cluster analysis) as-well-as categorical and continuous data [43, 44]. The two-step cluster analysis is most effective when all variables are independent, continuous variables are normally distributed, and categorical variables have a multinomial distribution [43, 44]. Cluster analysis still produces valid results even when cluster data do not meet the identified requirements for best practice because significance levels are not calculated as part of the test [43, 44].

The two-step clustering method uses a model-based measure of distance which defines the distance between two clusters prior to merging them [45]. Step 1 in the two-step cluster analysis involves pre-cluster formation to reduce the matrix size by minimizing distances between all pairs of cases [43, 44]. All cases in the same pre-cluster are treated as a single entity following this initial step [43, 44]. Step 2 of the two-step cluster analysis is a modified hierarchical agglomerative process where pre-clusters from Step 1 are combined sequentially, to form homogenous clusters [43, 44]. The two-step cluster method thus produces clusters through minimising within-cluster variance and maximizing between-cluster variance [43, 44]. The two-step cluster procedure provides an overall goodness-of-fit measure called silhouette measure of cohesion and separation [43, 44], which is based on average distances between objects and ranges from − 1 to + 1 [43, 44]. Silhouette measures of < 0.2 indicate poor cluster quality, measures of 0.2–0.5 indicate a fair cluster quality and measures > 0.5 indicate a good cluster quality [43, 44].

To ascertain that lifestyle risk factor clusters were different from each other, as cluster analysis does not involve hypothesis testing, clusters were compared using chi-squared tests, one-way analysis of variance (ANOVA) tests, or Welch ANOVA, depending on which test assumptions the different variables satisfied [43, 44]. Where results from the ANOVA were statistically significant, a Tukey HSD post hoc was used to assess specific differences between the lifestyle risk factors. Where the Welch ANOVA produced statistically significant results, the Games-Howell post hoc test was performed to assess specific differences between the lifestyle risk factors. Demographic variables of participants were also compared across the lifestyle risk factor clusters using chi-squared tests or ANOVA depending on suitability. Variables included in the demographic comparison of clusters were: sex, age in years, current marital status, highest qualification obtained, diagnosis and government pension as main source of income.

A statistical result was considered significant if *p* < 0.05.

## Results

### Sample characteristics (see Table 1)

Men comprised more than half of the sample (59.6%) with a mean participant age of 38 years (SD = 11.16). Only 4.9% from the cohort were of Aboriginal or Torres Strait Islander descent and most were single or never married (61.2%). Schizophrenia was the most prevalent diagnosis (47.0%) and most of the cohort (85.0%) received a government pension as the main source of income. A significant proportion of the sample (40.5%) had at least a tertiary certificate while more than half (59.2%) earned between $500 and $799 per fortnight. Most of the sample did not meet recommendations for vegetable intake (87.8%) or for fruit intake (71.1%). Almost one fifth of the cohort did not meet NHF’s target for HDL-C (22.5%) and over half did not meet the target for LDL-C (50.6%). An even greater proportion (64.8%) did not meet total cholesterol targets. Most participants were dyslipidaemic (70.7%) whereas about one-fifth displayed hyperglycaemia (21.8%). About one-third of the cohort (33.9%) had hypertension. Just under one-third of the cohort (31.6%) had moderate nicotine dependence with a slightly higher proportion being non-smokers (36.2%). The average vigorous and moderate physical activity undertaken per week was 150 min (SD = 354.3) and 120 min (SD = 383.6) respectively.

**Table 1:** Sociodemographic, Lifestyle and Metabolic Characteristics of Participants from the Survey of High Impact Psychosis

		*N* = 1825 (N %) M (SD)
Sex	Males	1087 (59.6%)
	Female	738 (40.4%)
Age	Mean	38.36 (11.16)
Aboriginal/ Torres Strait Islander descent	No	1735 (95.1%)
	Yes	90 (4.9%)
Marital Status	Single/ Never Married	1117 (61.2%)
	Married/ De facto	312 (17.1%)
	Separated/ Divorced	376 (20.6%)
	Widowed	20 (1.1%)
Diagnosis	Schizophrenia	857 (47.0%)
	Schizoaffective	293 (16.1%)
	Bipolar, mania	319 (17.5%)
	Depressive psychosis	81 (4.4%)
	Delusional disorders and other non-organic psychosis	92 (5.0%)
	Severe depression disorder	158 (8.7%)
	Screen-positive for psychosis but did not meet full criteria for ICD-10 psychosis	25 (1.4%)
Government pension, allowance or benefit as the main source of income	No	254 (13.9%)
	Yes	1551 (85.0%)
Highest qualification attained	Left school no qualifications	615 (33.7%)
	Secondary school qualification/leaving certificate	304 (16.7%)
	Tertiary Certificate	739 (40.5%)
	Bachelor’s Degree	92 (5.0%)
	Postgraduate Qualifications	28 (1.5%)
	Other	32 (1.8%)
	Missing	15 (0.8%)
Income per Fortnight	Less than $300 per fortnight	58 (3.2%)
	Between $300 - $499 per fortnight	209 (11.5%)
	Between $500 - $799 per fortnight	1080 (59.2%)
	Between $800 - $1000 per fortnight	232 (12.7%)
	More than $1000 per fortnight	139 (7.6%)
	Missing	107 (5.9%)
Vegetables consumed (no of serves per day in the last 4 weeks)	Met recommendations (≥4–5 serves/day)	206 (11.3%)
	Did not meet recommendations (≤0–3 serves/day)	1602 (87.8%)
	Missing	17 (0.9%)
Fruit consumed (no of serves per day in the last 4 weeks)	Met recommendations (≥2–3 serves/ day)	511 (28.0%)
	Did not meet recommendations (≤1 serve/ day)	1297 (71.1%)
	Missing	17 (0.9%)
HDL-C level	Mean	1.18 (0.42)
	Met target > 1 mmol/L	966 (52.9%)
	Did not meet target ≤1 mmol/L	411 (22.5%)
	Missing	448 (24.5%)
LDL-C level	Mean	3.13 (1.29)
	Met target < 2.5 mmol/L	326 (20.6%)
	Did not meet target ≥2.5 mmol/L	923 (50.6%)
	Missing	526 (28.8%)
Total Cholesterol	Mean	5.09 (1.19)
	Met target < 4 mmol/L	209 (11.5%)
	Did not meet target ≥4 mmol/L	1182 (64.8%)
	Missing	434 (23.8%)
Dyslipidaemia	Not Dyslipidemic	92 (5.0%)
	Dyslipidemic	1290 (70.7%)
	Missing	443 (24.3%)
Fasting plasma glucose	Mean	5.4 (1.21)
	Not hyperglycaemic (< 5.6 mmol/L)	990 (54.2%)
	Hyperglycaemic (≥5.6 mmol/L)	397 (21.8%)
	Missing	438 (24.0%)
Systolic blood pressure	Not hypertensive (< 140 mmHg)	1520 (83.3%)
	Hypertensive (≥140 mmHg)	246 (13.5%)
	Missing	59 (3.2%)
Diastolic blood pressure	Not hypertensive (< 90 mmHg)	1273 (69.8%)
	Hypertension (≥90 mmHg)	493 (27.0%)
	Missing	59 (3.2%)
Total Hypertensive	Not hypertensive (systolic < 140 mmHg or diastolic < 90 mmHg)	1207 (66.1%)
	Hypertensive (systolic ≥140 mmHg or diastolic ≥90 mmHg)	559 (30.6%)
	Missing	59 (3.2%)
Smoking- Fagerstrom test for nicotine dependence	Does not smoke	661 (36.2%)
	Low dependence	74 (4.1%)
	Low to moderate dependence	187 (10.2%)
	Moderate dependence	576 (31.6%)
	High dependence	327 (17.9%)
Time in min spent engaging in vigorous physical activity/ week	Mean	150.0 (354.3)
Time in min spent engaging in moderate physical activity/ week	Mean	120.0 (383.6)

ICD: International Statistical Classification of Diseases and Related Health Problems; M (SD) - mean (standard deviation). Missing data were either not provided by participants or could not be detected in the lab in the case of metabolic health measures

### Logistic regression modelling (see Tables 2, 3 and 4)

According to logistic regression analysis, associations of fruit intake, vegetable intake, total vigorous activity and total moderate activity with dyslipidaemia were not statistically significant. However, participants who did not smoke were about 50% less likely to have dyslipidaemia than those who smoked [Odds Ratio (OR) =0.54; 95% Confidence Interval (CI): 0.35–0.83; *p* = 0.005). Adjusting the analysis for clozapine use, sex and age resulted in little difference of the effect of smoking on dyslipidaemia (OR = 0.50; 95% CI: 0.32–0.78; *p* = 0.002) (Table 2).

**Table 2:** Logistic Regression Analysis of Smoking, Vegetable and Fruit Intake, and Physical Activity with Dyslipidaemia for Survey of High Impact Psychosis Participants

Independent Variables	Dependent Variable	Unadjusted Odds Ratio, (95% CI) and *p*-value	Adjusted Odds Ratio ^a^, (95% CI) and *p*-value
Smoking Status (^b^Non Smokers /Smokers)		0.54	0.50
		(0.35–0.83)	(0.32–0.78)
		0.005	0.002***
Vegetable intake (^b^Did not meet recommendations/ Met recommendations)		1.09	1.01
		(0.71–1.67)	(0.66–1.54)
		0.69	0.96
Fruit intake (^b^Did not meet recommendations/ Met recommendations)	Dyslipidaemia	1.06	1.12
		(0.71–1.67)	(0.77–1.65)
		0.79	0.54
Total vigorous activity in min/ week		1.00	1.00
		(1.00–1.00)	(1.00–1.00)
		0.04	0.10
Total moderate activity in min/ week		1.00	1.00
		(1.00–1.00)	(1.00–1.00)
		0.58	0.55

^a^ Adjusted for clozapine use, sex and age; ^b^ − reference value; ** *p* < 0.05; ***p < 0.01; ****p < 0.001; CI- Confidence interval

**Table 3:** Logistic Regression Analysis of Smoking, Vegetable and Fruit Intake, and Physical Activity with Hypertension for Survey of High Impact Psychosis Participants

Independent Variables	Dependent Variable	Unadjusted Odds Ratio, (95% CI) and p-value	Adjusted Odds Ratio ^a^, (95% CI) and *p*-value
Smoking Status (^b^Non Smokers /Smokers)		0.97	0.99
		(0.78–1.19)	(0.80–1.23)
		0.75	0.92
Vegetable intake (^b^Did not meet recommendations/ Met recommendations)	Hypertension	1.16	1.21
		(0.94–1.43)	(0.98–1.50)
		0.16	0.08
Fruit intake (^b^Did not meet recommendations/ Met recommendations)		0.95	0.89
		(0.78–1.15)	(0.73–1.09)
		0.60	0.26
Total vigorous activity in min/ week		1.00	1.00
		(1.00–1.00)	(1.00–1.00)
		0.68	0.71
Total moderate activity in min/ week		1.00	1.00
		(1.00–1.00)	(1.00–1.00)
		0.94	0.81

^a^ Adjusted for clozapine use, sex and age; ^b^ − reference value; ** *p* < 0.05; ***p < 0.01; ****p < 0.001; CI- Confidence interval

**Table 4:** Logistic Regression Analysis of Smoking, Vegetable and Fruit Intake, and Physical Activity with Hyperglycaemia for Survey of High Impact Psychosis Participants

Independent Variables	Dependent Variable	Unadjusted Odds Ratio, (95% CI) and *p*-value	Adjusted Odds Ratio ^a^, (95% CI) and *p*-value
Smoking Status (^b^Non Smokers /Smokers)		1.09	1.15
		(0.86–1.40)	(0.89–1.48)
		0.47	0.29
Vegetable intake (^b^Did not meet recommendations/ Met recommendations)		1.07	1.15
		(0.83–1.38)	(0.88–1.49)
		0.60	0.29
Fruit intake (^b^Did not meet recommendations/ Met recommendations)	Hyperglycaemia	1.17	1.04
		(0.89–1.40)	(0.83–1.31)
		0.33	0.73
Total vigorous activity in min/ week		1.00	1.00
		(1.00–1.00)	(1.00–1.00)
		0.038	0.16
Total moderate activity in min/ week		1.00	1.00
		(1.00–1.00)	(1.00–1.00)
		0.60	0.62

^a^ Adjusted for clozapine use, sex and age; ^b^ − reference value;** *p* < 0.05; ***p < 0.01; ****p < 0.001; CI- Confidence interval

Smoking, fruit intake, vegetable intake, total vigorous activity and total moderate activity were not significantly associated either with hypertension (Table 3) or hyperglycaemia (Table 4).

### Cluster analysis (see Table 5)

The two-step cluster analysis yielded three clusters with a silhouette measure of cohesion and separation of 0.5, indicating fair cluster quality. Cluster 1 (*n* = 401) had the fewest members and was characterised by the poorest lifestyle risk behaviours. All (100%) of cluster 1 members failed to meet recommendations for fruit or vegetable intake and had moderate dependence to nicotine. Total time per week spent engaging in vigorous physical activity was 22.5 min (SD = 80.2) whereas the total time spent engaging in moderate physical activity per week was 52 min (SD = 135.2). Cluster 2 (*n* = 771) differed from cluster 1 in that almost half of (*n* = 46.8%) cluster 2 members did not smoke, however, like cluster 1, almost all (97.8%) cluster 2 members failed to meet recommendations for fruit intake or for vegetable intake. Activity levels in cluster 2 were also low with the average time spent engaging in vigorous and moderate physical activity per week being 29 min (SD = 85.2) and 37.1 min (SD = 91.4) respectively. Cluster 3 (*n* = 653) was characterised by higher activity levels with average time spent engaging in vigorous activity and moderate physical activity being 107 min (SD = 297.3) and 136 min (SD = 368.3) respectively. More than three-quarters (78.3%) of cluster 3 members met fruit intake recommendations, in contrast to clusters 1 and 2.

**Table 5:** Cluster Analysis for Fruit Intake, Vegetable Intake, Smoking and Physical Activity in Survey of High Impact Psychosis Participants and Associated Demographic Profiles

Variables used for Cluster Formation		Cluster 1 n = 401 (22% of SHIP cohort) (N %) (poorest lifestyle behaviours)*	Cluster 2 n = 771 (42% of SHIP cohort) (N %) (mixture of poor and healthy lifestyle behaviours)*	Cluster 3 n = 653 (36% of SHIP cohort) (N %) (healthiest lifestyle behaviours)*	Statistical Test
Fruit consumed (no of serves per day in the last 4 weeks)	Met recommendations (≥2–3 serves/ day)	–	2.2	78.3	x^2^ (4) = 1288.69, p < 0.001****
	Did not meet recommendations (≤1 serve/ day)	100	97.8	21.7	
Vegetables consumed (no of serves per day in the last 4 weeks)	Met recommendations (≥4–5 serves/day)	–	2.2	31.5	x^2^ (4) = 436.39, p < 0.001****
	Did not meet recommendations (≤0–3 serves/day)	100	97.8	68.5	
Smoking- Fagerstrom test for nicotine dependence	Does not smoke	–	46.8	45.9	x^2^ (8) = 1257.55, p < 0.001****
	Low dependence	–	6.4	3.8	
	Low to moderate dependence	–	16.1	9.6	
	Moderate dependence	100	0.5	26.2	
	High dependence	–	30.2	14.4	
Time in min spent engaging in vigorous physical activity/ week		22.5 (80.2)^a^	29.0 (85.2)^b^	107.3 (297.3)^**c**^	(F (2, 994.5) = 23.4, p < 0.001****) GH ^a vs b^*p* = 0.355 ^b vs c^ p < 0.001**** ^a vs c^ p < 0.001****
Time in min spent engaging in moderate physical activity/ week		52 (135.2)^a^	37.1 (91.4)^b^	136.6 (368.3)^c^	(F (2, 833.3) = 24.1, p < 0.001****) GH ^a vs b^*p* = 0.088 ^b vs c^ p < 0.001**** ^a vs c^ p < 0.001****
Demographics Characteristics of Clusters					
Sex	Males	66.1	60.3	54.7	x^2^ (2) =13.75, p = 0.001***
	Females	33.9		45.3	
Age		36.77 (10.5)^a^	38.46 (11.5)^b^	39.23 (11.1)^c^	(F (2, 1798) = 5.9, p = 0.003***) T-HSD ^a vs b^ p = 0.019** ^b vs c^*p* = 0.160 ^a vs c^ p = 0.001***
Marital status	Single/ Never Married	66.3	59.7	59.9	x^2^ (6) = 7.35, *p* = 0.289
	Married/ De facto	15.2	18.0	17.2	
	Separated/ Divorced	17.7	21.4	21.4	
	Widowed	0.7	0.9	1.5	
Highest qualification obtained	Left school no qualifications	37.7	34.0	30.9	x^2^ (12) = 27.02, p = 0.008***
	Secondary school qualification/leaving certificate	17.7	15.2	17.8	
	Tertiary Certificate	39.2	39.6	42.4	
	Bachelor’s Degree	3.0	6.7	4.3	
	Postgraduate Qualifications	0.5	1.3	2.5	
	Other	1.5	2.3	1.2	
Diagnosis	Schizophrenia	48.9	47.9	44.7	x^2^ (30) = 30.66, *p* = 0.432
	Schizoaffective	17.2	14.6	17.1	
	Bipolar, mania	15.7	15.7	19.8	
	Depressive psychosis	3.5	5.4	3.8	
	Delusional disorders and other non-organic psychosis	1.2	5.1	4.5	
	Severe depression disorder	5.9	1.4	1.8	
	Screen-positive for psychosis but did not meet full criteria for ICD-10 psychosis	1.5	1.3	1.4	
Government pension, allowance or benefit as the main source of income	No	9.7	12.7	17.9	x^2^ (2) = 15.953, p < 0.001****
	Yes	89.8	86.1	80.7	

*clusters names are attributed to the most pronounced health behaviour characteristics in each cluster;** *p* < 0.05; ***p < 0.01; ****p < 0.001; SHIP- Survey of High Impact Psychosis; x^2^-Chi Square test; F- ANOVA- One-Way Analysis of Variance or Welch ANOVA; T-HSD- Tukey’s honestly significant difference post hoc analysis; GH- Games-Howell- post hoc analysis

When lifestyle risk factors were assessed for difference across different clusters, statistically significant results were obtained for all behaviours. There was a statistically significant difference in fruit intake across the clusters (x^2^ (4) = 1288.69, *p* < 0.001). All participants in cluster 1 (100%) failed to meet fruit intake recommendations, comparable to cluster 2 (97.8%), while in cluster 3, most met recommendations (78.3%). Similarly, vegetable intake differed across the clusters (x^2^ (4) = 436.39, *p* < 0.001): all participants in cluster 1 (100%) failed to meet recommendations for vegetable intake, comparable to cluster 2 (97.8%) participants whereas 68.5% of cluster 3 participants failed to meet recommendations.

Smoking rates as measured by the FTND differed between the clusters (x^2^ (8) = 1257.55, *p* < 0.001). All cluster 1 participants had a moderate dependence to nicotine (100%), compared with almost half of cluster 2 (46.8%) and cluster 3 (45.9%) participants, who did not smoke.

Time spent engaging in vigorous activity was significantly different across the clusters (F (2, 994.5) = 23.4, p < 0.001). Games-Howell post hoc analysis revealed statistically significant differences between clusters 2 and 3 (p < 0.001) and between clusters 1 and 3 (p < 0.001). Time spent engaging in moderate physical activity also differed across the clusters (F (2, 833.3) = 24.1, p < 0.001). Similarly, Games-Howell post hoc analysis also revealed statistically significant differences between clusters 2 and 3 (p < 0.001) and clusters 1 and 3 (p < 0.001). When participants’ demographic characteristics were compared across the three clusters, there was a significant association between cluster membership and sex (x^2^ (2) =13.75, *p* = 0.001). The largest proportion of men was in cluster 1 (66.1%), compared to cluster 2 (60.3%) and cluster 3 (54.7%).

There was also a significant difference in age of participants across the clusters (F (2, 1798) = 5.9, *p* = 0.003). Participants in cluster 1 were the youngest (36.77 ± 10.5) compared to those from cluster 2 (38.46 ± 11.5) and cluster 3 (39.23 ± 11.1). Tukey’s HSD post hoc analyses revealed that there were statistically significant differences between clusters 1 and 2 (*p* = 0.019) and clusters 1 and 3 (p = 0.001).

There was a statistically significant difference in the highest qualifications attained by participants across the clusters (x^2^ (12) = 27.02, *p* = 0.008). A smaller percentage of participants in clusters 1 and 2 had tertiary certificates (39.2 and 39.6% respectively) than in cluster 3 (42.4%). Finally, there was a statistically significant difference in those receiving a government pension as their main source of income across the clusters (x^2^ (2) = 15.953, *p* < 0.001). Cluster 1 had 89.8% of participants receiving a government pension as the main source of income whereas this percentage was 86.1% for cluster 2 and 80.7% for cluster 3.

## Discussion

The aim of this study was to describe the relationship between lifestyle risk factors for CVD, which are poor nutrition, smoking and low physical activity levels with dyslipidaemia, hypertension and hyperglycaemia in people living with psychosis while controlling for confounders. Smoking had a significant positive association with dyslipidaemia but showed no association with hypertension or hyperglycaemia. Fruit intake, vegetable intake, and physical activity were however not associated with dyslipidaemia, hypertension or hyperglycaemia. Clozapine use, sex and age were identified as potential confounding factors in the relationship between the lifestyle risk factors and dyslipidaemia, hypertension and hyperglycaemia, however their impact was marginal. This study also aimed to find clustering patterns of lifestyle risk factors and their associated demographic profiles and consequently identified three clusters.

Participants with who did not smoke were 50% less likely to have dyslipidaemia than those who smoked; no association was however found between fruit intake, vegetable intake, and physical activity levels with dyslipidaemia. The relationship between smoking and dyslipidaemia mirrors that of the general population [46–48]. However, research in the general population shows that consuming certain fruits and vegetables has a positive impact on markers of dyslipidaemia; nutrition tools used in our study were not specific to type of fruit or vegetables consumed, possibly explaining why our results did not reflect those from the general population [49–51]. Similarly, our results did not reflect the inverse relationship between physical activity levels and dyslipidaemia in the general population [52, 53]. Despite using a validated tool to measure physical activity levels, it is possible that factors such as participant bias, impaired memory, and confounding factors unique to this cohort impacted validity of the results in varying degrees [24, 25, 40, 42, 51, 54]. Future studies in people with psychosis can combat this by combining self-reported data with direct measures of physical activity levels [55, 56]. Lifestyle risk factors and unknown confounders are the likely cause of high rates of dyslipidaemia in people with psychosis thus, the use of lifestyle intervention efforts could be recommended [48–51].

We did not find any relationship between fruit and vegetable intake, smoking and physical activity levels with hypertension. Our results mirrored those in the general population except in the case of fruit intake and physical activity, which both have a negative relationship with hypertension [57–60]. Reasons for this are likely similar to those explained in the case of dyslipidaemia [56, 61].

Finally, fruit and vegetable intake, smoking and physical activity levels were not related to hyperglycaemia. In the general population, however, there is an inverse relationship between consumption of certain fruits and vegetables and the risk of type 2 diabetes [60]. Findings from the general population also confirm a positive relationship between smoking and risk of type 2 diabetes and a negative relationship between physical activity and type 2 diabetes risk [62, 63]. In addition to previously discussed limitations, discrepancy of our findings to those in the general population may exist because we used hyperglycaemia (≥5.6 mmol/L) to detect risk of type 2 diabetes; risk of type 2 diabetes considerably increases if fasting blood glucose is between 6.1–6.9 mmol/L or if plasma glucose is between 7.8–11.0 mmol/L based on a 2 h oral glucose tolerance test [10]. Uncontrolled hyperglycaemia can however still progress to type 2 diabetes, therefore, it is important that this risk is mitigated through lifestyle intervention [64].

Importantly, we showed that lifestyle risk factors occur in clusters among people with psychosis. The cluster with the highest prevalence of low fruit and vegetable intake, smoking and low physical activity had the highest proportion of men, who were younger, less likely to have a tertiary certificate and received a government pension as the main income. Research in the general population also found that younger men are most at risk for poor lifestyle risk behaviours [65, 66]. Younger unemployed men with psychosis are thus an important group to target in lifestyle interventions focusing all three lifestyle risk factors.

Participants in this study were most likely to belong to the cluster with low fruit and vegetable intake and low physical activity levels but least likely to smoke. This information highlights that people with psychosis mainly struggle with the nutrition and physical activity aspects of their lifestyle [15]. The demographic profile of this group appears to be mixed across age, gender, education and income. Nutrition and physical activity should thus be one of the primary focus areas of lifestyle interventions delivered to people with psychosis.

The final cluster in this cohort was characterised by a significant proportion of participants meeting fruit and vegetable recommendations, lower smoking rates and higher physical activity levels. This cluster had the highest proportion of women, showed a tendency towards increasing age, a higher proportion had tertiary certificates and fewer participants relied on government pensions as the main income. Similar demographic characteristics were found in the general population among those most likely to display healthy lifestyle behaviours [66, 67]. These results imply that socioeconomic or cognitive factors may be related to engagement with healthy lifestyle behaviours [66, 67]. The socioeconomic and cognitive profile of participants should therefore be considered during the conception of lifestyle interventions not only among people with psychosis but also in the general population so that solutions offered are viable for the target population [66, 67].

### Strengths and limitations

Due to the cross-sectional nature of SHIP, cause and effect relationships cannot be confirmed from our results, therefore, longitudinal research should be carried out to broaden findings from the current study [15]. SHIP is however to date the largest, representative study of people with psychosis in Australia [15]. SHIP covered a broad range of topics (in a long interview with the potential to lead to participant fatigue), thus, direct, detailed and longitudinal information on nutrition, smoking, physical activity and metabolic health could not be obtained [15]. To ameliorate this however, trained interviewers with excellent inter-rater reliability administered SHIP, using validated tools in the measurement of nutrition, smoking, physical activity, diagnosis, and metabolic health, minimizing bias [15].

## Conclusions

In summary, smoking status had a significant positive association with dyslipidaemia but showed no relationship with hypertension and hyperglycaemia [38]. Fruit and vegetable intake and physical activity were however not associated with dyslipidaemia, hypertension and hyperglycaemia, which in some cases contradicted previous research [56, 61, 68]. Although this was the case, previous literature from the general population gives us reason to recommend lifestyle interventions as the primary prevention for CVD among people with psychosis [17]. Additionally, clozapine use, sex and age did not seem to have a significant impact in the relationship between lifestyle risk factors and dyslipidaemia, hypertension and hyperglycaemia - hence other confounding factors need to be identified and their impact quantified. Finally, lifestyle risk factors for CVD occurred in three main clusters with characteristic demographic profiles which could be useful for the design and implementation of lifestyle intervention programs in people with psychosis.

## Abbreviations

ADGs: Australian Dietary Guidelines
BMI: Body mass index
CMOs: Community-managed organisations
CVD: Cardiovascular disease
DIP-DM: Diagnostic Module of the Diagnostic Interview for Psychosis
FTND: Fagerstrom Test for Nicotine Dependence
HDL-C: High-density lipoprotein cholesterol
ICD: International Statistical Classification of Diseases and Related Health Problems
IPAQ: International Physical Activity Questionnaire
LDL-C: Low-density lipoprotein cholesterol
MHSs: Mental health services
NHF: National Heart Foundation
NHMRC: The National Health and Medical Research Council
NNS: National Nutrition Survey
SHIP: Survey of High Impact Psychosis
SPSS: Statistical Package for the Social Sciences
SPSS: Statistical Package for the Social Sciences

## References

[1] World Health Organization*.* About cardiovascular diseases. [Internet ] 2017 [cited 2017 March 14]; Available from: http://www.who.int/cardiovascular_diseases/about_cvd/en/.

[2] Mozaffarian D, Benjamin EJ, Go AS, Arnett DK, Blaha MJ, Cushman M, Das SR, de Ferranti S, Després J-P, Fullerton HJ. Heart disease and stroke statistics—2016 update. Circulation. 2016, 133(4):e38-e360. 10.1161/CIR.0000000000000350 26673558

[3] Laursen TM, Munk-Olsen T, Vestergaard M (2012). Life expectancy and cardiovascular mortality in persons with schizophrenia. Curr Opin Psychiatry.

[4] Kritharides L, Chow V, Lambert TJ (2017). Cardiovascular disease in patients with schizophrenia. Med J Aust.

[5] Galletly CA, Foley DL, Waterreus A, Watts GF, Castle DJ, McGrath JJ, Mackinnon A, Morgan VA. Cardiometabolic risk factors in people with psychotic disorders: the second Australian national survey of psychosis. Aust N Z J Psychiatry. 2012:0004867412453089. 10.1177/0004867412453089 22761397

[6] Connolly M, Kelly C (2005). Lifestyle and physical health in schizophrenia. BJPsych Advances.

[7] Mendis, S.;P. Puska, and B. Norrving. Global atlas on cardiovascular disease Prev Control [Internet ] 2011 [cited 2017 May 28]; Available from: http://www.who.int/cardiovascular_diseases/publications/atlas_cvd/en/.

[8] Alberti KGM, Zimmet P, Shaw J (2005). The metabolic syndrome-a new worldwide definition. Lancet.

[9] Tonkin A, Barter P, Best J, Boyden A, Furler J, Hossack K, Sullivan D, Thompson P, Vale M, Cooper C (2005). National Heart Foundation of Australia and the Cardiac Society of Australia and New Zealand: position statement on lipid management--200*5*. Heart Lung Circ.

[10] World Health Organization. Definition and diagnosis of diabetes mellitus and intermediate hyperglycemia: Report of a WHO consultation. Geneva: World Health Organization [Internet ] 2006 [cited 2017 March 17]; Available from: http://www.who.int/diabetes/publications/Definition%20and%20diagnosis%20of%20diabetes_new.pdf.

[11] National Heart Foundation of Australia. Guidelines for the diagnosis and management of hypertension in adults [Internet ] 2016 [cited 2017 March 29]; Available from: https://www.heartfoundation.org.au/images/uploads/publications/PRO-167_Hypertension-guideline-2016_WEB.pdf.

[12] Australian Institute of Health and Welfare. Risk factors to health, Insufficient physical activity. [Internet ] 2017 [cited 2017 June 13]; Available from: https://www.aihw.gov.au/reports/biomedical-risk-factors/risk-factors-to-health/contents/insufficient-physical-activity.

[13] National Health and Medical Research Council. Eat for Health, Australian Dietary Guidelines. 2013 [cited 2016 October 10]; Available from: https://www.eatforhealth.gov.au/sites/default/files/files/the_guidelines/n55_australian_dietary_guidelines.pdf.

[14] Haddad PM, Sharma SG (2007). Adverse effects of atypical antipsychotics. CNS drugs.

[15] Morgan VA, Waterreus A, Jablensky A, Mackinnon A, McGrath JJ, Carr V, Bush R, Castle D, Cohen M, Harvey C (2012). People living with psychotic illness in 2010: the second Australian national survey of psychosis. Aust N Z J Psychiatry.

[16] Alberti K, Eckel RH, Grundy SM, Zimmet PZ, Cleeman JI, Donato KA, Fruchart JC, James WPT, Loria CM, Smith SC (2009). Harmonizing the metabolic syndrome a joint interim statement of the international diabetes federation task force on epidemiology and prevention; national heart, lung, and blood institute; American heart association; world heart federation; international atherosclerosis society; and international association for the study of obesity. Circulation.

[17] Mozaffarian D, Wilson PW, Kannel WB (2008). Beyond established and novel risk factors lifestyle risk factors for cardiovascular disease. Circulation.

[18] Diabetes Prevention Program Research Group (2002). Reduction in the incidence of type 2 diabetes with lifestyle intervention or metformin. N Engl J Med.

[19] Chiuve SE, McCullough ML, Sacks FM, Rimm EB (2006). Healthy lifestyle factors in the primary prevention of coronary heart disease among men: benefits among users and nonusers of lipid-lowering and antihypertensive medications. Circulation.

[20] Osborn DP, Nazareth I, King MB (2007). Physical activity, dietary habits and coronary heart disease risk factor knowledge amongst people with severe mental illness. Soc Psychiatry Psychiatr Epidemiol.

[21] Osborn DP, Hardoon S, Omar RZ, Holt RI, King M, Larsen J, Marston L, Morris RW, Nazareth I, Walters K (2015). Cardiovascular risk prediction models for people with severe mental illness: results from the prediction and management of cardiovascular risk in people with severe mental illnesses (PRIMROSE) research program. JAMA psychiatry.

[22] Foley DL, Mackinnon A, Morgan VA, Watts GF, Shaw JE, Magliano DJ, Castle DJ, McGrath JJ, Waterreus A, Galletly CA (2015). Cardiovascular risk factor associations in adults with psychosis and adults in a national comparator sample. Aust N Z J Psychiatry.

[23] Correll CU, Solmi M, Veronese N, Bortolato B, Rosson S, Santonastaso P, Thapa-Chhetri N, Fornaro M, Gallicchio D, Collantoni E (2017). Prevalence, incidence and mortality from cardiovascular disease in patients with pooled and specific severe mental illness: a large-scale meta-analysis of 3,211,768 patients and 113,383,368 controls. World Psychiatry.

[24] Morgan V, McGrath J, Jablensky A, Badcock J, Waterreus A, Bush R, Carr V, Castle D, Cohen M, Galletly C (2014). Psychosis prevalence and physical, metabolic and cognitive co-morbidity: data from the second Australian national survey of psychosis. Psychol Med.

[25] Foley DL, Mackinnon A, Watts GF, Shaw JE, Magliano DJ, Castle DJ, McGrath JJ, Waterreus A, Morgan VA, Galletly CA (2013). Cardiometabolic risk indicators that distinguish adults with psychosis from the general population, by age and gender. PLoS One.

[26] Foley DL, Mackinnon A, Morgan VA, Watts GF, McGrath JJ, Castle DJ, Waterreus A, Galletly CA (2014). Predictors of type 2 diabetes in a nationally representative sample of adults with psychosis. World Psychiatry.

[27] Mucheru D, Hanlon M-C, Campbell LE, McEvoy M, MacDonald-Wicks L (2017). Social dysfunction and diet outcomes in people with psychosis. Nutrients.

[28] Noble N, Paul C, Turon H, Oldmeadow C (2015). Which modifiable health risk behaviours are related? A systematic review of the clustering of smoking, nutrition, alcohol and physical activity (‘SNAP’) health risk factors. Prev Med.

[29] Nigg CR, Allegrante JP, Ory M (2002). Theory-comparison and multiple-behavior research: common themes advancing health behavior research. Health Educ Res.

[30] Goldstein MG, Whitlock EP, DePue J, P.C.O.T.A.M.B.R.F.I.P.C. Project (2004). Multiple behavioral risk factor interventions in primary care: summary of research evidence. Am J Prev Med.

[31] Bonfioli E, Berti L, Goss C, Muraro F, Burti L (2012). Health promotion lifestyle interventions for weight management in psychosis: a systematic review and meta-analysis of randomised controlled trials. BMC psychiatry.

[32] Hahn LA, Galletly CA, Foley DL, Mackinnon A, Watts GF, Castle DJ, Waterreus A, Morgan VA (2014). Inadequate fruit and vegetable intake in people with psychosis. Aust N Z J Psychiatry.

[33] Suetani S, Waterreus A, Morgan V, Foley D, Galletly C, Badcock J, Watts G, McKinnon A, Castle D, Saha S (2016). Correlates of physical activity in people living with psychotic illness. Acta Psychiatr Scand.

[34] Lv J, Liu Q, Ren Y, Gong T, Wang S, Li L (2011). Socio-demographic association of multiple modifiable lifestyle risk factors and their clustering in a representative urban population of adults: a cross-sectional study in Hangzhou, China. Int J Behav Nutr Phys Act.

[35] Mucheru DW, Hanlon MC, McEvoy M, MacDonald-Wicks L (2017). Comparative efficacy of lifestyle intervention strategies on weight outcomes in people with psychosis: a systematic review and network meta-analysis protocol. JBI Database System Rev Implement Rep.

[36] Castle DJ, Jablensky A, McGrath JJ, Carr V, Morgan V, Waterreus A, Valuri G, Stain H, McGuffin P, Farmer A (2006). The diagnostic interview for psychoses (DIP): development, reliability and applications. Psychol Med.

[37] Bergman H, Khodabakhsh A, Maayan N, Kirkham AJ, Adams CE, Soares-Weiser K. Operational criteria checklist for psychotic illness and affective illness (OPCRIT+) for diagnosing schizophrenia in people with psychotic symptoms. Cochrane Libr. 2014;

[38] Garriguet D (2009). Diet quality in Canada. Health Rep.

[39] Rutishauser, I.H.;K. Webb;B. Abraham, and R. Allsopp. Evaluation of short dietary questions from the 1995 National Nutrition Survey. Canberra: Australian Government Department of Health and Ageing 2001 [cited 2017 27 May]; Available from: https://www.health.gov.au/internet/main/publishing.nsf/Content/17B241DC3956A205CA257BF00020A773/$File/evaluation.pdf.

[40] Weinberger AH, Reutenauer EL, Allen TM, Termine A, Vessicchio JC, Sacco KA, Easton CJ, McKee SA, George TP (2007). Reliability of the fagerström test for nicotine dependence, minnesota nicotine withdrawal scale, and tiffany questionnaire for smoking urges in smokers with and without schizophrenia. Drug Alcohol Depend.

[41] National Institute of Drug Abuse. *Fagerstrom Score*. [cited 2016 10th August]; Available from: http://cde.drugabuse.gov/instrument/d7c0b0f5-b865-e4de-e040-bb89ad43202b/module/f7cc1db9-2f13-70d5-e040-bb89ad4345a3/question/f7cc1db9-2f15-70d5-e040-bb89ad4345a3.

[42] Faulkner G, Cohn T, Remington G (2006). Validation of a physical activity assessment tool for individuals with schizophrenia. Schizophr Res.

[43] Sarstedt M, Mooi E (2014). *A concise guide to market research*. 2nd ed ed. the process, data, and using IBM SPSS statistics.

[44] Yim O, Ramdeen KT (2015). Hierarchical cluster analysis: comparison of three linkage measures and application to psychological data. Quant Methods Psychol.

[45] Meilă M, Heckerman D (2001). An experimental comparison of model-based clustering methods. Mach Learn.

[46] Craig WY, Palomaki GE, Haddow JE (1989). Cigarette smoking and serum lipid and lipoprotein concentrations: an analysis of published data. BMJ.

[47] Masulli M, Vaccaro O (2006). Association between cigarette smoking and metabolic syndrome. Diabetes Care.

[48] Haj Mouhamed D, Ezzaher A, Neffati F, Gaha L, Douki W, Najjar MF (2013). Association between cigarette smoking and dyslipidemia. IMMUNO ANAL BIOL SPE.

[49] Basu A, Fu DX, Wilkinson M, Simmons B, Wu M, Betts NM, Du M, Lyons TJ (2010). Strawberries decrease atherosclerotic markers in subjects with metabolic syndrome. Nutr Res.

[50] Erlund I, Koli R, Alfthan G, Marniemi J, Puukka P, Mustonen P, Mattila P, Jula A (2008). Favorable effects of berry consumption on platelet function, blood pressure, and HDL cholesterol. Am J Clin Nutr.

[51] MacDonald AW, Schulz SC (2009). What we know: findings that every theory of schizophrenia should explain. Schizophr Bull.

[52] Thompson PD, Buchner D, Piña IL, Balady GJ, Williams MA, Marcus BH, Berra K, Blair SN, Costa F, Franklin B (2003). Exercise and physical activity in the prevention and treatment of atherosclerotic cardiovascular disease. Arterioscler Thromb Vasc Biol.

[53] Myers J (2003). Exercise and cardiovascular health. Circulation.

[54] De Hert M, Dekker J, Wood D, Kahl K, Holt R, Möller H-J (2009). Cardiovascular disease and diabetes in people with severe mental illness position statement from the European psychiatric association (EPA), supported by the European Association for the Study of diabetes (EASD) and the European Society of Cardiology (ESC). Eur Psychiatry.

[55] Blank MD, Disharoon S, Eissenberg T (2009). Comparison of methods for measurement of smoking behavior: mouthpiece-based computerized devices versus direct observation. Nicotine Tob Res.

[56] Prince SA, Adamo KB, Hamel ME, Hardt J, Gorber SC, Tremblay M (2008). A comparison of direct versus self-report measures for assessing physical activity in adults: a systematic review. Int J Behav Nutr Phys Act.

[57] Berglund G, Wilhelmsen L (1975). Factors related to blood pressure in a general population sample of Swedish men. J Intern Med.

[58] Primatesta P, Falaschetti E, Gupta S, Marmot MG, Poulter NR (2001). Association between smoking and blood pressure. Hypertension.

[59] Li B, Li F, Wang L, Zhang D (2016). Fruit and vegetables consumption and risk of hypertension: a meta-analysis. J Clin Hypertens.

[60] Yurong Z, Liwei C, Gang H (2011). Physical activity and hypertension: results from cohort studies and meta-analysis. J Educ Res.

[61] Souverein OW, de Vries JHM, Freese R, Watzl B, Bub A, Miller ER, Castenmiller JJM, Pasman WJ, Van Het Hof K, Chopra M (2015). Prediction of fruit and vegetable intake from biomarkers using individual participant data of diet-controlled intervention studies. Br J Nutr.

[62] Pan A, Wang Y, Talaei M, Hu FB, Wu T (2015). Relation of active, passive, and quitting smoking with incident type 2 diabetes: a systematic review and meta-analysis. Lancet Diabetes Endocrinol.

[63] Jeon CY, Lokken RP, Hu FB, Van Dam RM (2007). Physical activity of moderate intensity and risk of type 2 diabetes. Diabetes Care.

[64] Bruins J, Jörg F, Bruggeman R, Slooff C, Corpeleijn E, Pijnenborg M (2014). The effects of lifestyle interventions on (long-term) weight management, cardiometabolic risk and depressive symptoms in people with psychotic disorders: a meta-analysis. PLoS One.

[65] Tobias M, Jackson G, Yeh LC, Huang K (2007). Do healthy and unhealthy behaviours cluster in New Zealand?. Aust N Z J Public Health.

[66] French S, Rosenberg M, Knuiman M (2008). The clustering of health risk behaviours in a western Australian adult population. Health Promot J Austr.

[67] Conry MC, Morgan K, Curry P, McGee H, Harrington J, Ward M, Shelley E (2011). The clustering of health behaviours in Ireland and their relationship with mental health, self-rated health and quality of life. BMC Public Health.

[68] Van de Mortel TF (2008). Faking it: social desirability response bias in self-report research. Australian Journal of Advanced Nursing, The.

